# CDK Inhibition Primes for Anti-PD-L1 Treatment in Triple-Negative Breast Cancer Models

**DOI:** 10.3390/cancers14143361

**Published:** 2022-07-11

**Authors:** Anthony Cheung, Alicia M. Chenoweth, Jelmar Quist, Heng Sheng Sow, Christina Malaktou, Riccardo Ferro, Ricarda M. Hoffmann, Gabriel Osborn, Eirini Sachouli, Elise French, Rebecca Marlow, Katie E. Lacy, Sophie Papa, Anita Grigoriadis, Sophia N. Karagiannis

**Affiliations:** 1 St. John’s Institute of Dermatology, School of Basic & Medical Biosciences, King’s College London, Guy’s Hospital, London SE1 9RT, UK; anthony.cheung@kcl.ac.uk (A.C.); alicia.chenoweth@kcl.ac.uk (A.M.C.); hengsheng.sow@kcl.ac.uk (H.S.S.); c.malaktou19@imperial.ac.uk (C.M.); ricarda.hoffmann@icr.ac.uk (R.M.H.); gabriel.osborn@kcl.ac.uk (G.O.); eirini.sachouli17@imperial.ac.uk (E.S.); elise.french@babraham.ac.uk (E.F.); katie.lacy@gstt.nhs.uk (K.E.L.); 2 Breast Cancer Now Research Unit, School of Cancer & Pharmaceutical Sciences, King’s College London, Guy’s Cancer Centre, London SE1 9RT, UK; jelmar.quist@kcl.ac.uk (J.Q.); riccardo.ferro@kcl.ac.uk (R.F.); rebecca.marlow@icr.ac.uk (R.M.); anita.grigoriadis@kcl.ac.uk (A.G.); 3 ImmunoEngineering, School of Cancer and Pharmaceutical Sciences, Faculty of Life Sciences and Medicine, King’s College London, London SE1 9RT, UK; sophie.papa@kcl.ac.uk

**Keywords:** triple-negative breast cancer, CDK2, cyclin E, checkpoint immunotherapy, PD-L1, avelumab, cell cycle, cyclin-dependent kinases

## Abstract

**Simple Summary:**

The cyclin E/CDK2 complex may present a promising target axis for the treatment of triple-negative breast cancers (TNBC); however, therapeutically relevant doses of CDK2 inhibitors have been associated with toxicities. Here, we report that the suboptimal dosing of the CDK 2, 7 and 9 inhibitor SNS-032 reduced the viability of TNBC cells and upregulated the checkpoint ligand PD-L1 expression in surviving cancer cells in vitro and in human orthotopic MDA-MB-231 TNBC xenografts grown in immunodeficient mice. Moreover, in immunodeficient, TNBC xenograft-bearing mice engrafted with human immune cells, SNS-032 treatment was associated with the infiltration of CD45^+^ human immune cells in tumors. In these orthotopic MDA-MB-231 TNBC-bearing mice, suboptimal SNS-032 doses given sequentially ahead of dosing with the anti-PD-L1 antibody avelumab significantly restricted tumor growth compared with monotherapy. These findings suggest that surviving cancer cells following suboptimal CDK inhibitor treatment may be responsive to checkpoint immunotherapy.

**Abstract:**

Triple-negative breast cancers (TNBC) expressing PD-L1 qualify for checkpoint inhibitor immunotherapy. Cyclin E/CDK2 is a potential target axis in TNBC; however, small-molecule drugs at efficacious doses may be associated with toxicity, and treatment alongside immunotherapy requires investigation. We evaluated CDK inhibition at suboptimal levels and its anti-tumor and immunomodulatory effects. Transcriptomic analyses of primary breast cancers confirmed higher cyclin E/CDK2 expression in TNBC compared with non-TNBC. Out of the three CDK2-targeting inhibitors tested, the CDK 2, 7 and 9 inhibitor SNS-032 was the most potent in reducing TNBC cell viability and exerted cytotoxicity against all eight TNBC cell lines evaluated in vitro. Suboptimal SNS-032 dosing elevated cell surface PD-L1 expression in surviving TNBC cells. In mice engrafted with human immune cells and challenged with human MDA-MB-231 TNBC xenografts in mammary fat pads, suboptimal SNS-032 dosing partially restricted tumor growth, enhanced the tumor infiltration of human CD45^+^ immune cells and elevated cell surface PD-L1 expression in surviving cancer cells. In tumor-bearing mice engrafted with human immune cells, the anti-PD-L1 antibody avelumab, given sequentially following suboptimal SNS-032 dosing, reduced tumor growth compared with SNS-032 alone or with avelumab without prior SNS-032 priming. CDK inhibition at suboptimal doses promotes immune cell recruitment to tumors, PD-L1 expression by surviving TNBC cells and may complement immunotherapy.

## 1. Introduction

Triple-negative breast cancers (TNBC), forming the majority of the PAM50 criteria-classified basal-like cancer subtype, are defined by a lack of estrogen receptor (ER), progesterone receptor (PR) and human epidermal growth factor receptor 2 (HER2) expression. TNBC comprise a heterogeneous disease group associated with increased genomic instability and high mitotic rates [1,2]. Historically, while few targeted therapies have been available for these patients, recently, the anti-programmed death-ligand 1 (PD-L1) antibody immunotherapy atezolizumab in combination with nab-paclitaxel chemotherapy received regulatory approval for the treatment of advanced-stage TNBC [3,4]. However, several studies have also reported a substantial heterogeneity of PD-L1 expression in TNBC lesions [5,6,7], and only patients with tumors that express PD-L1 are eligible to receive this immunotherapy.

The binding of PD-L1 expressed by cancer cells to programmed death receptor-1 (PD-1) in immune cells is thought to inhibit effector T lymphocyte function and limit immune system-mediated tumor cell destruction [8,9]. Over 200 clinical trials were opened to breast cancer patients to assess drugs targeting the PD-/PD-L1 axis, either as monotherapy or in combination with other regimens [10]. The results of immunotherapy in TNBC show modest response rates ranging from 5% to 22% with anti-PD-1 and anti-PD-L1 monoclonal antibody monotherapies, such as pembrolizumab and avelumab, respectively [11,12,13,14]. However, a combination of chemotherapy, irradiation or targeted therapy together with anti-PD-L1 immunotherapy demonstrated some success, likely due to beneficial immunomodulatory effects stimulated by anti-tumor chemotherapy agents [3,4,15,16,17]. PD-L1 expression can be controlled at both transcriptional and post-translational levels, and studies suggested that PD-L1 stability depends on physiological conditions. PD-L1 expression fluctuates during the cell cycle progression in several human cancers, including in TNBC, with higher levels reported to be expressed in mitotic and early G1 phases [18]. It is therefore possible that interfering with cancer cell signaling may also influence PD-L1 expression.

The dysregulation of the cell cycle can support breast cancer growth, and the blockade of cyclin-dependent kinase (CDK)4/6 with the inhibitor palbociclib has demonstrated a clinical benefit for advanced ER+ breast cancer [19]. By inhibiting kinase activity, CDK4/6 inhibitors are able to block cell-cycle progression from phase G1 to phase S and prevent the progression of cancer cells [20]. It has been reported that PD-L1 abundance may also be regulated by cyclin D/CDK4, and inhibitors of CDK4/6 can increase PD-L1 expression [18]. This may point to the potential of evaluating CDK4/6 inhibitors with immunotherapy targeting the PD-1/PD-L1 axis [21,22]. However, most TNBC are resistant to CDK4/6 inhibition, and resistance has been shown to result from the compensatory upregulation of other kinases such as CDK2 [23,24]. CDK2 becomes activated upon binding to cyclin E. The cyclin E/CDK2 complex formation promotes the G1-to-S-phase cell cycle transition by phosphorylating RB and reducing the inhibition of the transcription factor E2F [25]. Although CDK2 is a potential target for therapy in TNBC, selective CDK2 inhibitors are still in preclinical development, while promiscuous CDK inhibitors often display toxicity in early clinical trials [26]. Therefore, treatment strategies, including combinations that require lower inhibitor doses may be required. Since CDK4/6 inhibitors have been shown to influence PD-L1 protein levels and enhance immune cell infiltration [18], here we evaluated whether CDK2 inhibition in TNBC cells at suboptimal doses may have immunomodulatory effects and be combined with immunotherapy.

## 2. Materials and Methods

### 2.1. Gene Expression Data from Human Breast Cancers

Detailed descriptions of METABRIC cohort (*n* = 1096), The Cancer Genome Atlas (TCGA) Breast cohort (*n* = 578) and TNBC-enriched King’s College London Guy’s Hospital cohort (Guy’s) (*n* = 177) were previously reported [27,28,29,30,31]. Normal breast tissue was available for TCGA (*n* = 112), collected > 2 cm from the breast tumor margin and without tumor as assessed by histopathology, and Guy’s (*n* = 10) was collected from reduction mammoplasty. A collection of publicly available primary breast cancer and normal tissue cohorts are summarized in Suppl. Table S1. Clinicopathological and gene expression data were extracted from publications and compared between immunohistochemistry (IHC)-defined subtypes that are based on routine breast pathology evaluation of ER, PR and HER2 receptors, or compared between PAM50 subtypes that are based on the expression of 50 genes to classify breast cancers into five distinct subtypes (Basal-like, HER2-enriched, Luminal A, Luminal B and Normal-like) [2,32,33]. All statistical analysis and respective data plots were generated in the R environment, using several CRAN packages (http://cran.rproject.org/ accessed on 1 April 2022).

Differential expression profiles were plotted using the publicly available GEPIA dataset (http://gepia.cancer-pku.cn/ accessed on 1 April 2022), which included *n* = 1085 breast tumors and *n* = 291 normal breast samples [34] (Suppl. Table S1). *CDK2* gene expression in eight TNBC cell line models were analyzed using publicly available online database Cancer Cell Line Encyclopedia (CCLE) (https://portals.broadinstitute.org/ccle accessed on 1 April 2022) [35]. CIBERSORT was used for the immune cell analysis of the Guy’s cohort gene expression data [36].

### 2.2. Immunohistochemical Protein Profiling Data

CDK2 and cyclin E distribution and relative protein expression levels in 24 normal human organs (derived from immunohistochemical staining of tissue sections from one to three healthy volunteer donors per organ type) were evaluated from a publicly available tissue database based on antibody proteomics (The Human Protein Atlas, version 19.3) (all images are available on www.proteinatlas.org accessed on 1 April 2022) [37] (Suppl. Table S1). Identical immunohistochemical staining was performed on tumor samples; however, breast cancer subtypes were not specified in this database.

### 2.3. Breast Cell Lines

All cell lines were obtained from King’s College London Breast Cancer Now Unit, except HDQ-P1, purchased from Leibniz Institute DSMZ. Cell lines were authenticated by short tandem repeat profiling. Cells used once tested negative for mycoplasma and used up to 30 passages. All cell lines were maintained in 5% CO_2_ humidified incubator at 37 °C.

### 2.4. Human Peripheral Blood Mononuclear Cell (PBMC) and NK Cell Isolation

Healthy volunteer peripheral blood samples were collected with informed written consent, in accordance with the Helsinki Declaration. Study design was approved by the Guy’s Research Ethics Committee, Guy’s and St. Thomas’ NHS Foundation Trust. Peripheral blood was also obtained through the UK National Health Service (NHS) Blood and Transplant Service from anonymous donor leukocyte cones. PBMCs were isolated using Ficoll Paque PLUS (Cytiva, Marlborough, MA, USA) density gradient centrifugation. Red blood cells were lysed from extracted PBMCs using RBC Lysis buffer (Biolegend, London, UK). NK cells were isolated using the RosetteSep™ Human NK Cell Enrichment Cocktail (STEMCELL™ Technologies, Cambridge, UK).

### 2.5. In Vitro Cell Viability Assay

To compare the potency of CDK inhibitors to impair cell viability, breast cancer cell lines were plated on 96-well plates and treated with various concentrations of palbociclib (Merck Life Science Limited, Dorset, UK), K03861 (MedChemexpress LLC., Monmouth Junction, NJ, USA), JNJ-7706621 (Biovision, Oxfordshire, UK) or SNS-032 (Tocris Bioscience, Bristol, UK). DMSO was used as vehicle control. Cell viability was measured by the methyl tetrazolium (MTT) assay (Promega, Southampton, UK) as per manufacturers’ protocol. Optical absorbance was read on FLUOstar Omega spectrophotometer (BMG Labtech, Buckinghamshire, UK) to determine viable cell counts compared with controls after 96 h.

### 2.6. IncuCyte Live-Imaging Analysis

Cell lines were treated with SNS-032 (0.1 and 0.2 μM) and live phase-contrast images (×20 magnification) were taken using an IncuCyte® S3 Zoom Live-Imaging system (Essen Bioscience, Hertfordshire, UK) for a 72 h period to determine the effect on cell growth. Cell count was measured using the IncuCyte® S3 software (version 2019A). Ethidium homodimer-1 (Thermo Fisher Scientific) (4 μM), which is incorporated into the DNA of dead cells, was added to inhibitor-treated cells to determine the effect of SNS-032 or palbociclib (0.1 μM) on cell death. Red fluorescence signals were monitored for 72 h. The effect of avelumab (10 μg/mL) on NK cell-mediated cytotoxicity in mCherry-transfected TNBC cells (ratio: one cancer cell to four purified NK cells) was measured by IncuCyte Zoom Live-Imaging. The mCherry signals were monitored for 72 h to determine the growth of TNBC cells.

### 2.7. Flow Cytometric Analysis of PD-L1 Expression

Breast cancer cells were treated with DMSO or SNS-032 (0.2 μM) for 48 h. To measure PD-L1 expression levels in vitro, cells were detached, and direct immunofluorescence staining was performed for 20 min on ice using FITC-conjugated anti-human CD274 (PD-L1) antibody (Biolegend, London, UK). DAPI (Life Technologies, Paisley, UK) staining was used for the exclusion of dead cells. Samples were acquired using the FACSCanto™ II flow cytometer with BD FACSDiva Software (BD Biosciences, Franklin Lakes, NJ, USA) and analyzed with FlowJo_V10 software.

### 2.8. In Vivo Xenograft Studies

Six-week-old female NSG mice were used and handled in accordance with Institutional Committees on Animal Welfare (The Home Office Animals Scientific Procedures Act, 1986). Animals were orthotopically injected in the mammary fat pad with 1 × 10^6^ cancer cells in 50 μL PBS mixed in 50 μL Matrigel (Day 1). Tumors were measured with calipers, and volumes were calculated as π × length × width^2^/6. To test PD-L1 expression in vivo in response to SNS-032 treatment, a small single dose of SNS-032 (15 mg/kg), or equivalent 0.75 g/kg DMSO was intravenously injected in 200 μL volume to the tail veins when tumors reached 8 mm × 8 mm (measured using a caliper) (i.e., 266 mm^3^). Tumors were harvested 3 days post-injection for FACS analysis of PD-L1 expression. Sequential combination therapy experiments were initiated once palpable tumors were formed, of 3 mm × 3 mm in size (i.e., 14 mm^3^). Once tumors reached the minimum size required, mice received an intravenous injection of SNS-032 (15 mg/kg) (Day 6), followed by anti-PD-L1 antibody avelumab (Bavencio) (10 mg/kg) pre-mixed with a single dose of 10 × 10^6^ human healthy volunteer PBMC to provide effector cells (Day 9). Subsequent inhibitor doses (Day 13, 20, 27) and antibody doses (Day 16, 23, 30) were given weekly. Experiments terminated after 34 days, with tumor sizes no greater than 525 mm^3^. Human immune cell engraftment in mouse tissues was calculated using the formula: human CD45^+^ cells/(human CD45^+^ cells + mouse CD45^+^ cells) × 100%. PD-L1 expression by non-immune cells (human CD45^−^ mouse CD45^−^ cells) was also confirmed by flow cytometry. Flow cytometric gating excluded any dead cells and cell aggregates which could increase autofluorescence and non-specific antibody staining. Antibodies used were rat anti-mouse CD45-V500 (BD Biosciences, Franklin Lakes, NJ, USA), mouse anti-human CD45-PE-Cy7 (BD Biosciences, Franklin Lakes, NJ, USA) and FITC-conjugated anti-human CD274 (PD-L1) antibody (Biolegend, London, UK).

### 2.9. Statistical Analyses

The Cox proportional hazards regression model (Hazard Ratio (HR)) was used to test the statistical independence and significance in predicting the risk of breast cancer-specific death. Mann–Whitney U test was used for unpaired determination of overall immune infiltration correlated with *CDK2*/*CCNE1* expression. GraphPad Prism software was used for statistical analyses. All assay conditions were tested in triplicates. Data were presented as mean ± standard error of the mean (SEM). Kaplan–Meier overall survival plots were generated using an external cohort by online estimator (http://kmplot.com/analysis/ accessed on 1 April 2022) (KM plotter cohort, Suppl. Table S1) of 1402 breast cancer samples [38]. *p*-values were reported with the following associated symbols: *p* < 0.05 (*), *p* < 0.005 (**), *p* < 0.0005 (***), and all tests were two-sided.

## 3. Results

### 3.1. The Cyclin E/CDK2 Partners Are Upregulated in Basal-like/TNBC

**Figure 1:**
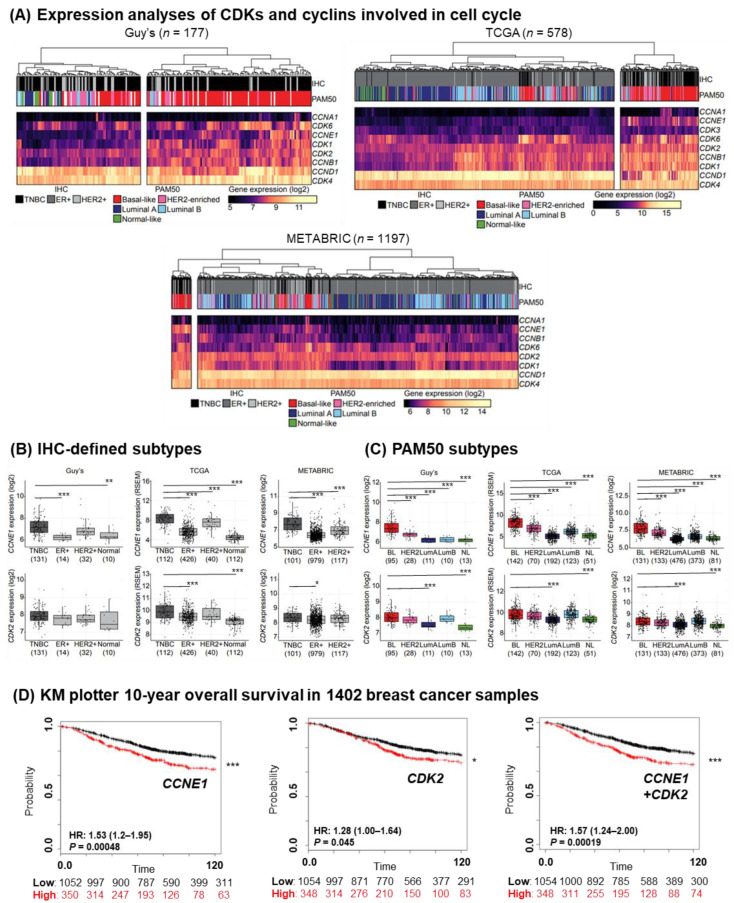
Basal-like/TNBC is associated with an upregulated expression level of the cyclin E/CDK2 partner. (**A**) Gene expression analysis of primary tumors from Guy’s (TNBC-enriched, *n* = 177), TCGA (*n* = 578) and METABRIC (*n* = 1197) cohorts of CDK and cyclin genes involved in cell cycle. Color scale indicates log2 expression values (yellow, higher; black, lower expression). Cohorts were divided into TNBC, ER+ and HER2+ groups based on their IHC-defined receptor status, in addition to PAM50 classification (Basal-like, HER2-enriched, luminal A, luminal B and normal-like). (**B**) *CCNE1* and *CDK2* expression levels were stratified according to their IHC-defined receptor status, or (**C**) PAM50 classification (Basal-like (BL), HER2, luminal A (LumA), luminal B (LumB) and normal-like (NL)). Median-centered gene expression log2 values are shown. Numbers of patients per group are indicated below the graphs. *p*-values were determined using a Mann–Whitney U test. (**D**) Kaplan–Meier curves shows the association of *CCNE1* and *CDK2* expression (upper quartile (red line) compared to the other curves (black line)) with ten-year overall survival in 1402 breast cancer samples. Significant *p*-values are indicated with an asterisk, whereas * *p* < 0.05; ** *p* < 0.005; *** *p* < 0.0005.

Since TNBC are known to be highly proliferative, we analyzed the gene expression of CDKs and cyclins involved in the cell cycle and transcription regulation from TNBC-enriched King’s College London Guy’s Hospital (Guy’s) cohort, containing primary breast cancers with no prior therapy at diagnosis (*n* = 177), in addition to the publicly available datasets (TCGA cohort (*n* = 578) and METABRIC cohort (*n* = 1096)) [27,28,29,30,31] (patient cohorts summarized in Suppl. Table S1). In heat maps of relative gene expression, samples were stratified by as previously described immunohistochemistry (IHC)-defined receptor status of ER, PR and HER2 expression, or by the PAM50 molecular classification (five intrinsic subtypes: luminal A (LumA), luminal B (LumB), HER2-enriched, basal-like, and normal-like breast cancer [2]) (Figure 1A). For the early G1-to-S-phase cell cycle genes, *CDK6* and *CCNE1* both showed a strong association with TNBC and basal-like breast cancers in all three cohorts, while *CDK2*, *CDK4* and *CCND1* expression were high across all patient samples. CDK4/6 are known targets of the inhibitor palbociclib, which is approved for the treatment of hormone receptor-positive breast cancer, while basal-like/TNBC are largely resistant [23]. There were no clear clustering of the transcriptional CDKs and cyclins that are associated with mediator complex and transcription factor regulation [39] (Suppl. Figure S1).

We confirmed that the gene expression of both cyclin E (encoded by *CCNE1*) and its binding partner *CDK2*, thought of as an escape mechanism to CDK4/6 inhibition [23], were upregulated in TNBC compared with ER+ or HER2+ tumors in samples stratified by IHC-defined receptor status (ER, PR and HER2 expression) (Figure 1B), in concordance with previous studies (METABRIC) [23] (although the difference in *CDK2* expression between TNBC and other subtypes did not reach significance in the Guy’s dataset, possibly due to the smaller sample number). When tumor lesions were stratified by PAM50 molecular classification, *CCNE1* expression levels were significantly higher in basal-like breast cancer compared with other molecular subtypes (Figure 1C). *CDK2* gene expression was also higher in basal-like subtype compared with the less aggressive LumA or normal-like breast tumors (Figure 1C). In addition, cyclin A, another binding partner of CDK2, was upregulated in basal-like/TNBC, together with the two kinases, CDK4 and CDK6, known to be crucial in cell cycle regulation (Suppl. Figure S2, gene expression data from TCGA and Guy’s cohorts). The ten-year overall survival of patients with primary breast cancers with high expression (defined as that of the upper quartile of the cohort) of cyclin E and CDK2 was significantly lower than for patients whose cancer lesions showed medium/low expression (publicly available KM plotter cohort, Suppl. Table S1, *n* = 1402) (Hazard Ratio (HR) = 1.53, *p* < 0.001 for cyclin E; HR = 1.28, *p* = 0.045 for CDK2; HR = 1.57, *p* < 0.001 for cyclin E/CDK2 partner) (Figure 1D).

These findings confirm a higher expression of the cyclin E/CDK2 partners in TNBC and an association of higher expression levels with less favorable outcomes in breast cancer.

### 3.2. TNBC Cell Targeting with CDK Inhibitors

**Figure 2:**
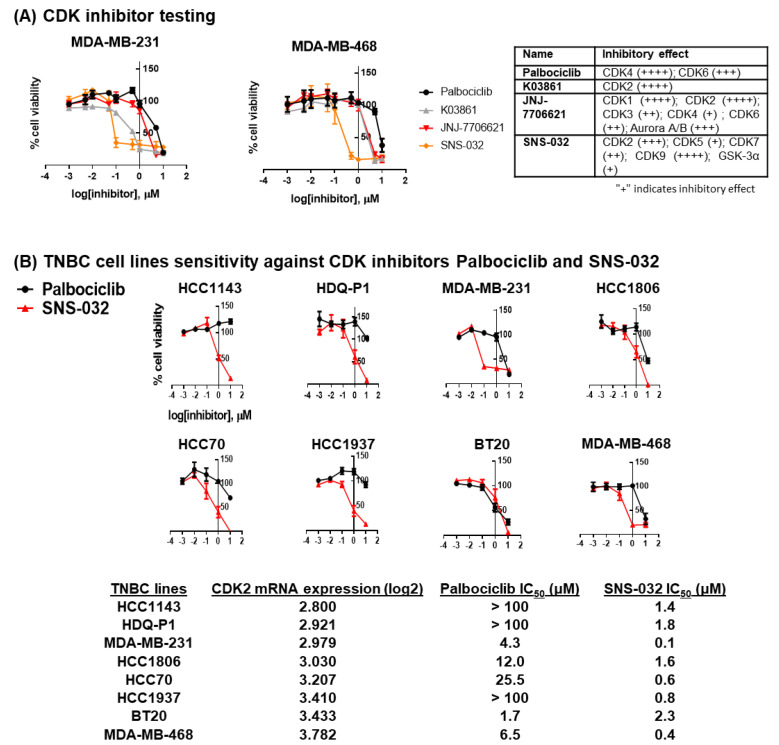
TNBC cell lines are sensitive to CDK inhibitor SNS-032. (**A**) Three small-molecule CDK inhibitors were tested on TNBC cell lines MDA-MB-231 and MDA-MB-468. Cells were treated with a gradient concentration of CDK inhibitors, and cell viability was tested using MTT assay (*n* = 3). Inhibitory effects of palbociclib [45], K03861 [40], JNJ-7706621 [41] and SNS-032 [42,43] measured in published cell-free assays are showed in the table, whereas increased inhibition is marked by a higher “+” designation. (**B**) A panel of eight TNBC cell lines were tested against the sensitivity of SNS-032 treatment (*n* = 3 for each cell line), compared to CDK4/6 inhibitor palbociclib and IC_50_ values were calculated (the Cancer Cell Line Encyclopedia (CCLE) database was used to characterize mRNA expression of *CDK2*). With the exception of BT20, TNBC cells were shown to be more resistant to palbociclib compared to the low half-maximal inhibitory concentration (IC_50_) for SNS-032 in all cell lines.

Based on high levels of cyclin E and CDK2 in breast cancers (Suppl. Figure S3, Gene Expression Profiling Interactive Analysis ((GEPIA) cohort [34] and Human Protein Atlas cohort [37], Suppl. Table S1), especially in TNBC, and since a CDK2-specific inhibitor is not yet publicly available, we tested in vitro three CDK inhibitors with high potency against CDK2, as opposed to potent and specific CDK4/6 inhibition. In agreement with previous findings [23], the TNBC cell lines MDA-MB-231 and MDA-MB-468 required high concentrations of palbociclib (close to 10 µM) to inhibit cell viability, indicating a level of resistance to CDK4/6 inhibition. The reduction in cell viability was evident following treatment with three tested pan-CDK inhibitors, namely K03861 [40], JNJ-7706621 [41] and SNS-032 [42,43]. Each one was previously reported to have high potency against CDK2 (Figure 2A, Table on the right) and studied in clinical trials in patients with solid tumors [42,43,44]. SNS-032 required the lowest concentrations (near 0.1 µm) to induce an effect (Figure 2A). SNS-032 was originally designed to be a selective CDK2 inhibitor [43], but has also been reported to block RNA Polymerase II activity by inhibiting CDK7 and CDK9 [42]. However, our data indicate that these two CDKs and their binding partners (cyclin H and cyclin T1) are downregulated in basal-like/TNBC (Suppl. Figure S4, gene expression data from TCGA, Guy’s and GEPIA cohorts).

**Figure 3:**
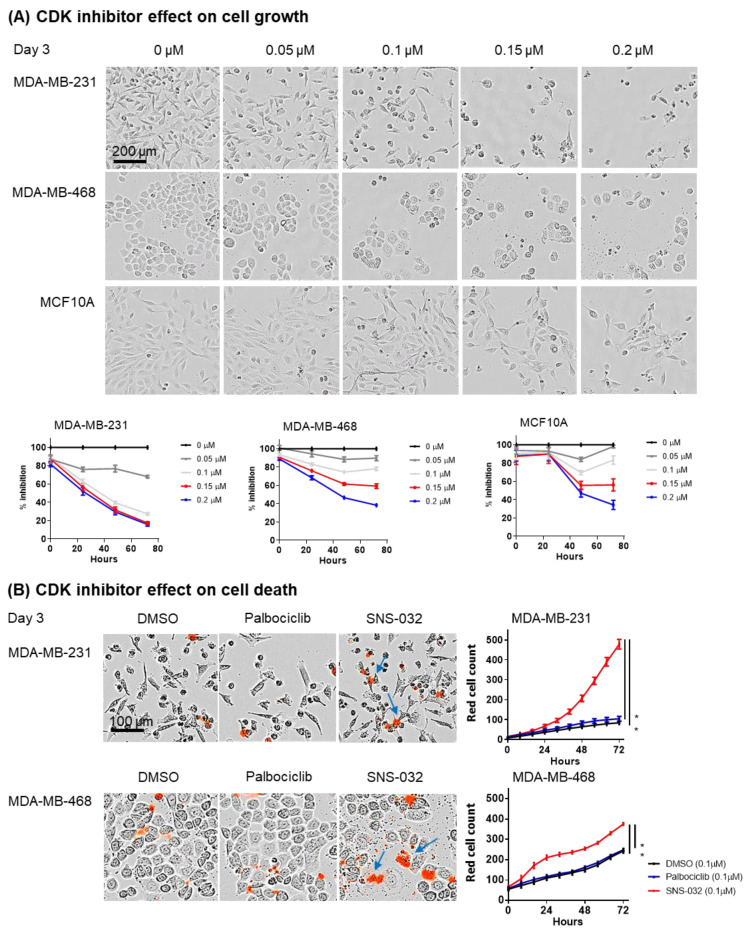
SNS-032 effect on cell growth and cell death by IncuCyte Zoom Live-Imaging analysis. (**A**) live phase-contrast images were taken using the IncuCyte Zoom Live-Imaging system to determine the effect of SNS-032 (0.1 and 0.2 μM) on cell growth over a 72 h period (*n* = 3). Scale bar of representative images = 200 μm. (**B**) Red fluorescence dead cell marker ethidium homodimer-1 was added to inhibitor-treated cells to determine the effect of SNS-032 or palbociclib (0.1 μM) on cell death over a 72 h period (*n* = 3). Scale bar of representative images = 100 μm. Significant restrictions in cellular growth and increases in cell death were shown in TNBC cell lines with SNS-032 treatment. All *p*-values were reported with the following associated symbols: *p* < 0.05 (*), and all tests were two-sided.

We subsequently evaluated the sensitivity of eight TNBC cell lines to a gradient concentration of SNS-032, compared to control CDK4/6-targeting palbociclib. With the exception of BT20, all TNBC cell lines were resistant to palbociclib but sensitive to SNS-032, with MDA-MB-231 demonstrating the lowest IC_50_ dose for inhibiting cancer cell viability (Figure 2B). In live-cell imaging studies (IncuCyte Zoom Live-Imaging), we monitored the growth rate of the cells using SNS-032 doses based on the IC_50_ dose evaluated in Figure 2. The data suggested that normal breast cell line MCF10A required a higher inhibition dosage than the TNBC cell lines, with little cell growth inhibition over a 72 h period with 0.1 µM SNS-032 (83.4 +/− 4.6% growth rate, normalized against the maximum cell count of the control untreated well, in comparison to 27.4 +/− 1.1% for MDA-MB231 and 74.1 +/− 3.6% for MDA-MB-468). A growth rate of 44 +/− 2.5% was measured for MCF10A cells at higher SNS-032 dose at 0.2 µM, compared to 15.9 +/− 1.7% for MDA-MB231 and 38.4 +/− 0.7% for MDA-MB-468 (Figure 3A). Quantitative analyses of the red fluorescence signal (ethidium homodimer-1) to identify dead cells also demonstrated cell death with SNS-032 treatment over a three-day culture (Figure 3B).

These findings suggest that basal-like/TNBC cells may be susceptible to inhibition by SNS-032 in vitro.

### 3.3. Evidence of PD-L1 Upregulation by SNS-032 Treatment In vitro and in TNBC Xenografts In Vivo

**Figure 4:**
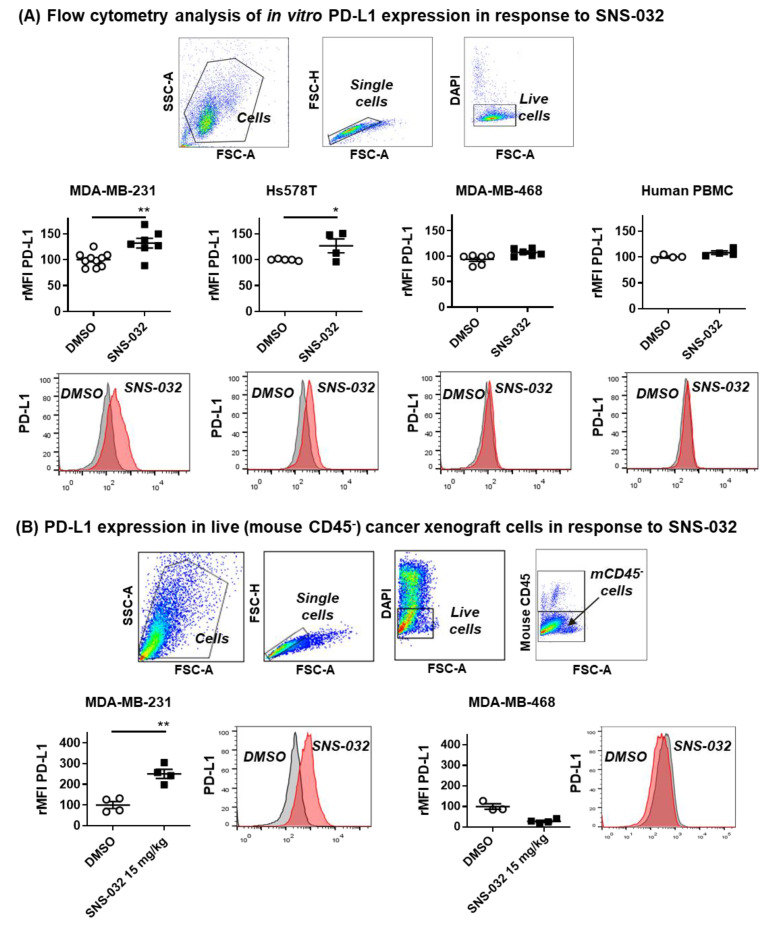
Upregulation of PD-L1 in cell culture and in TNBC xenograft models after SNS-032 treatment. (**A**) TNBC cell lines were treated with 0.2 μM SNS-032 or DMSO for 48 h and in vitro PD-L1 protein expression levels in live cancer cells were measured by flow cytometry (Top panel: rMFI; Bottom panel: histograms, grey: isotype control; pink: PD-L1) (*n* = 3). (**B**) Flow cytometric data analyses demonstrating elevated PD-L1 expression after SNS-032 treatment (15 mg/kg) in live ex vivo MDA-MB-231 cells (*n* = 4 mice each), but not in MDA-MB-468 model (*n* = 3 mice for DMSO group, *n* = 4 mice for SNS-032-treated group) Top panel: Flow cytometric gating strategy to select live non-immune cells extracted from human TNBC xenograft models; Bottom panel: PD-L1 expression by rMFI and histograms (grey: isotype control; pink: PD-L1). All *p*-values were reported with the following associated symbols: *p* < 0.05 (*), *p* < 0.005 (**), and all tests were two-sided.

Given the reported dependency of the cell cycle status for PD-L1 expression (see above), we next evaluated whether SNS-032 inhibition upregulated PD-L1 expression on TNBC cells. We studied cell surface PD-L1 expression by flow cytometric analyses of live TNBC cells, which survived 48 h of suboptimal SNS-032 treatment (based on the IC_50_ dose evaluated in Figure 2). We observed higher PD-L1 expression in those live MDA-MB-231 and Hs578T cancer cells after treatment with SNS-032. However, MDA-MB-468 cancer cells that expressed low levels of PD-L1 at baseline did not upregulate PD-L1 following SNS-032 treatment (Figure 4A). As positive controls, we confirmed that, similar to SNS-032 treatment, MDA-MB-231 and Hs578T, but not MDA-MB-468, demonstrated PD-L1 upregulation after treatment for 48 h, with IFN-γ, a well-described stimulant of PD-L1 expression [46] (Suppl. Figure S5A). This suggested that MDA-MB-231 and Hs578T were amenable to stimulation to enhance PD-L1, while MDA-MB-468 cells were resistant to PD-L1 upregulation by the known stimulant IFN-γ and by SNS-032, potentially reflecting the heterogeneity of PD-L1 expression in TNBC.

We next conducted flow cytometric analyses of tumor cells extracted from a human MDA-MB-231 TNBC xenograft grown in mouse mammary fat pads after systemic treatment of mice with suboptimal doses of SNS-032 (15 mg/kg intravenous dosing once weekly, compared with 15–30 mg/kg intraperitoneally every 3 days, as previously reported [47,48]). In concordance with our in vitro findings, these analyses demonstrated the elevated expression of human PD-L1 by the remaining live MDA-MB-231 cancer cells (mouse CD45^−^, DAPI^−^ fraction, using a detecting antibody recognizing only human, but not mouse, PD-L1). On the other hand, similarly to our in vitro findings, live cancer cells from MDA-MB-468 TNBC xenografts were expressed near background levels of PD-L1 without and following treatment with SNS-032 in vivo (Figure 4B).

In vitro cellular and in vivo xenograft models demonstrated evidence of PD-L1 upregulation by the remaining live TNBC cells following SNS-032 treatment.

### 3.4. Combination of SNS-032 and Anti-PD-L1 Sequential Therapy Restricted the Growth of Orthotopically Grown TNBC in Mice Engrafted with Human Immune Cells

Since MDA-MB-231 TNBC cells showed sensitivity to SNS-032 inhibition and an upregulation of PD-L1 by the remaining live cancer cells, we hypothesized that the dead cell debris generated from SNS-032 inhibition could activate anti-tumor immune surveillance and trigger lymphocyte recruitment, whereas PD-L1 upregulation on the surviving TNBC cells could offer a chance of treatment alongside immunotherapy. Therefore, we tested the susceptibility of MDA-MB-231 tumors to anti-PD-L1 antibody immunotherapy in vitro, and in combination with SNS-032 in vivo in orthotopically grown human TNBC xenografts in mammary fat pads of immunodeficient mice engrafted with human immune cells.

The increased expression of PD-L1 by tumor cells may make them susceptible to antibody-dependent cell-mediated cytotoxicity (ADCC) by human immune effector cells when stimulated by an IgG1 subclass antibody that has an active Fc region, such as avelumab [49]. We confirmed that avelumab could trigger human NK cell-mediated cancer cell cytotoxicity above controls (Suppl. Figure S5B) in cell viability assays in vitro. We measured viable cancer cell counts of PD-L1-expressing MDA-MB-231 cells (mCherry-transfected) for up to 72 h in co-culture assays together with human NK cells with or without avelumab (IncuCyte Zoom Live-Imaging tracking). Avelumab treatment in the presence of FcR-expressing effector cells (NK cells) restricted the growth of MDA-MB-231 cells above controls, while no cytotoxic effects were measured on the PD-L1-negative MDA-MB-468 cells.

Both MDA-MB-231 and Hs578T TNBC cell lines were susceptible to SNS-032 inhibition and the remaining live cancer cells upregulated PD-L1 following SNS-032 treatment in vitro. However, only MDA-MB-231 cell implantation in the mammary fat pad consistently resulted in the formation of tumor xenografts in vivo. MDA-MB-231 is a widely described TNBC model for evaluating novel drugs, including in the local disease setting [28,32,50]. We thus selected the MDA-MB-231 cancer cell model to examine the potential in vivo anti-tumor growth effects of SNS-032 together with avelumab in orthotopically grown human TNBC xenografts in mammary fat pads of immunodeficient mice complemented with human immune cells. These mice were deficient in mouse B cell, T cell and natural killer (NK) cell functions and major histocompatibility complex (MHC) class I/II expression, thus they were better able to tolerate human tumor xenograft growth and human immune cell engraftment [51,52].

**Figure 5:**
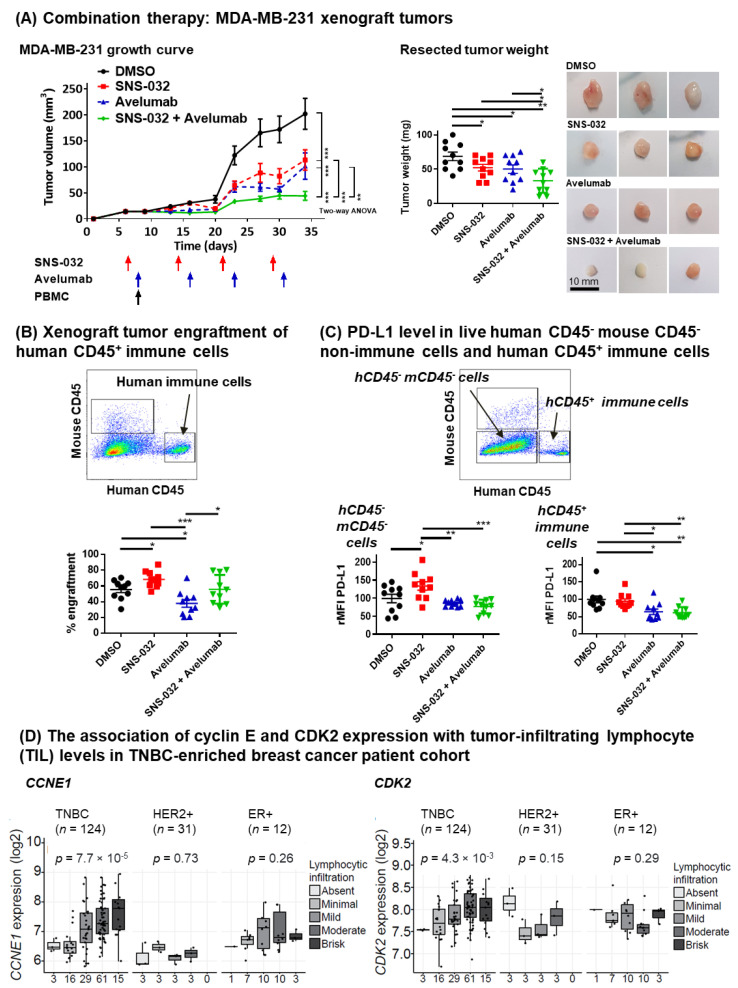
Combination treatment of SNS-032 with anti-PD-L1 avelumab antibody. (**A**) Tumor growth measurements following inoculation (**left**), resected tumor weights (**middle**) and representative tumor photographs (**right**) of MDA-MB-231 tumors of the partly immuno-humanized mice treated with DMSO, SNS-032 inhibitor alone, avelumab antibody alone, or with sequential dosing with SNS-032 and avelumab (*n* = 10 mice per condition). Once palpable tumors were formed, mice received an intravenous injection of SNS-032 (15 mg/kg) (on Day 6), followed by avelumab (10 mg/kg) pre-mixed with a single dose of human healthy volunteer PBMC to provide effector cells (on Day 9). Subsequent inhibitor doses (Day 13, 20, 27) and antibody doses (Day 16, 23, 30) were given weekly. (**B**) Human immune cell engraftment in mouse tissues (% engraftment) was calculated using the formula: human CD45^+^ cells/(human CD45^+^ cells + mouse CD45^+^ cells) × 100%. (**C**) PD-L1 expression levels of live non-immune cells (human and mouse CD45^−^ cells) (**left**) and live human CD45^+^ immune cells (**right**) were measured by flow cytometry with anti-human CD274 staining of extracted xenograft tumors. All *p*-values were reported with the following associated symbols: *p* < 0.05 (*), *p* < 0.005 (**), *p* < 0.0005 (***), and all tests were two-sided. (**D**) CIBERSORT was used for the immune cell analysis of the Guy’s cohort gene expression data. Lymphocytic infiltration levels were classified into five groups: absent, minimal, mild, moderate and brisk. Numbers of patients per group are indicated below the graphs. *p*-values were determined using Mann–Whitney U test for the CIBERSORT data.

As we previously reported [53], mice were challenged with human tumors in the mammary fat pads and human peripheral blood mononuclear cells (PBMC) were also engrafted to serve as FcR-expressing effector cells in order to evaluate human immune cell infiltration of tumors and anti-PD-L1 antibody effector functions in this model. We injected a relatively small amount of tumor cells (1 × 10^6^ cells) [54,55] aiming to monitor the growth of tumors throughout sequential combination treatments. Compared with vehicle treatment controls (Figure 5A, black line), SNS-032-alone (at a weekly dosage of 15 mg/kg; Figure 5A, red line) and avelumab-alone (at weekly dosage of 10 mg/kg, Figure 5A, blue line) treatments each resulted in partial tumor growth reduction. Sequential dosing of SNS-032 followed by avelumab treatment was associated with significantly reduced tumor growth (Figure 5A, left, green line) and weight (Figure 5A, middle: weights; right: representative images) compared with each monotherapy (*n* = 10 mice per group for all groups). Body weight measurements of mice, which received orthotopic TNBC cells and human PBMC and were treated with SNS-032, avelumab, or a combination, recorded no significant weight loss following the treatments (*n* = 37 out of 40 mice showed < 10% weight loss until the end of planned experiments), indicating a minimal graft-versus-host effect (Suppl. Figure S5C).

Human immune cell infiltration of tumors was confirmed by flow cytometric assessments of human CD45^+^ cells extracted from the human TNBC xenografts (Figure 5B). This demonstrated that SNS-032-alone treatment was associated with a higher total human (CD45^+^) immune cell engraftment compared with no treatment controls, and sequential combination treatment was associated with higher human CD45^+^ immune cell infiltration compared with avelumab alone. This may point to an immune surveillance response activated with SNS-032 inhibition. Further flow cytometric analyses revealed an infiltration of different immune cell populations (Suppl. Figure S6). A significant proportion of infiltrating CD45^+^ human cells recruited consisted of T cells. In avelumab and sequential combination treatment groups we measured a significant decrease in CD4 T cells, likely contributed by reduced T helper cell subpopulations. This is consistent with previous reports demonstrating that avelumab treatment on human PBMC results in a reduction in CD4 T lymphocyte proliferation (measured by Ki67) and a switch towards Th1 immune responses [56]. We also found a proportional reduction in NK cell infiltrates in the sequential combination therapy group, a key effector cell population for the IgG1 subclass avelumab antibody to mediate ADCC [49]. This may be associated with the stimulation and potential exhaustion following multiple SNS-032 priming and subsequent avelumab dosing and requires further investigation.

Furthermore, in single-tumor-cell extracts from TNBC xenografts, human PD-L1 expression was detected in non-mouse and non-immune cells (mouse CD45^−^ and human CD45^−^) with a detecting antibody recognizing only human, but not mouse, PD-L1 (Figure 5C, red dots). This confirmed our in vitro and in vivo findings (see Figure 4). Together, these findings suggest that following SNS-032 treatment, surviving human tumor cells showed increased cell surface PD-L1 expression. We previously measured a modest increase in PD-L1 expression by live (non-immune) human cancer cells in the SNS-032-alone-treated mice without human PMBC engraftment (see Figure 4B, MDA-MB-231 model). Here, in mice engrafted with human PBMC we detected no significant increase in PD-L1 expression (FITC-conjugated anti-human CD274) in human cancer cells from mice given avelumab alone or SNS-032 plus avelumab (Figure 5C). This is likely due to the avelumab-dependent loss of PD-L1-expressing tumor cells after four weeks of anti-PD-L1 treatment in vivo. Contrary to the increased PD-L1 expression by live non-immune cells (mouse CD45^−^ and human CD45^−^) with SNS-032 treatment (Figure 5C, left), surface PD-L1 expression by human CD45^+^ immune cells [57] was not affected by SNS-032 inhibition. PD-L1 expression by human CD45^+^ cells was reduced in the avelumab and combination treatment groups (Figure 5C, right). These findings suggest that: CDK inhibition enhanced PD-L1 expression by cancer cells but not by immune cells, and that PD-L1-expressing human immune cells may be depleted with anti-PD-L1 therapy.

In patients, avelumab might disrupt the PD-1-PD-L1 axis to reverse immunosuppression and might stimulate immune effector cells to trigger ADCC of PD-L1 expressing cancer cells via Fc-FcR interactions. We further tested the level of tumor-infiltrating lymphocyte (TIL) by CIBERSORT [36] in the TNBC-enriched Guy’s cohort, and we investigated their association with cyclin E and CDK2 expression (Figure 5D). TNBC samples with a high *CCNE1* or *CDK2* level were more likely to feature an immune-rich tumor microenvironment (TME) with a significant level of lymphocytic infiltration (moderate to brisk level). These potential immune effector cells could have roles in treatment response with the combination of SNS-032 and anti-PD-L1 therapy.

**Figure 6:**
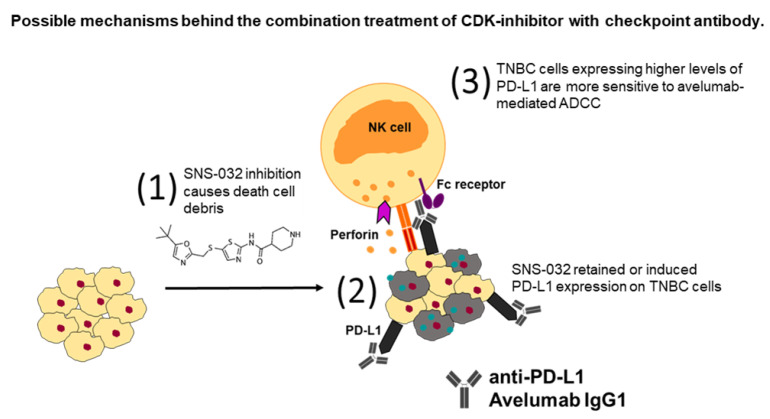
A model depicting possible mechanisms behind the combination treatment of small-molecule CDK inhibitors with anti-PD-L1 checkpoint antibody. (**1**) It is thought that treatment with CDK inhibitor SNS-032 led to the dysregulation of cell cycle and generated cytotoxicity against tumor cells, whereas death cell debris (grey cells and green dots) activated anti-tumor immune surveillance and triggered the recruitment of lymphocytes into the tumor microenvironment. (**2**) SNS-032 inhibition upregulated PD-L1 expression on the surviving TNBC cells which could increase susceptibility to checkpoint inhibition. Treatment with avelumab resulted in blockade of the PD-1/PD-L1 axis between immune cells and cancer cells. (**3**) Avelumab enhanced NK-cell-mediated perforin-dependent cytotoxicity, initiated by the ligation of Fc receptors from avelumab-bound tumor cells to FcγR expressed on NK cells, which led to tumor cell apoptosis. TNBC cells expressing higher levels of PD-L1 are more sensitive to avelumab-mediated ADCC. A combination of CDK inhibition with anti-PD-L1 checkpoint blockade could promote immune cell recruitment into tumors and lead to restriction of tumor growth.

Together, these findings suggest that suboptimal SNS-032 treatment was associated with: a) the enhanced infiltration of human CD45^+^ immune cells to mammary fat pad-grown MDA-MB-231 human TNBC xenografts in mice, which were systemically engrafted with human immune cells, and b) elevated cell surface PD-L1 expression by surviving TNBC cells in vivo (see Figure 6 for schematic of proposed mechanisms of combination therapy). Suboptimal SNS-032 dosing given prior to anti-PD-L1 antibody in tumor-bearing mice engrafted with human immune cells led to the restriction of tumor growth compared with avelumab without prior SNS-032 priming or SNS-032 alone.

## 4. Discussion

Studies in several in vitro and in vivo cancer models have demonstrated that the CDK4/6 inhibitor palbociclib can stimulate PD-L1 expression on cancer cells and induce immune cell infiltration in the TME [18,21]. In this study, having confirmed that the gene expression of the cyclin E/CDK2 partners may be upregulated in basal-like/TNBC cells and could be targeted with small-molecule inhibitors in vitro and in vivo, we provide evidence that treatment with the CDK inhibitor SNS-032, with high affinity for CDK2 among other kinases, may upregulate PD-L1 expression by surviving cancer cells in some TNBC in vitro cellular and in vivo xenograft models. In immunodeficient mice challenged with human TNBC cells in the mammary fat pad and engrafted with human immune cells, suboptimal SNS-032 treatment was associated with human immune cell infiltration into tumor lesions. In the same model, human TNBC xenograft-bearing mice given suboptimal weekly doses of SNS-032 prior to an anti-PD-L1 antibody showed restricted growth of mammary fat pad tumors.

We confirmed the expression of genes involved in cell cycle by cross-referencing to a study by Malumbres et al. [39] and the dysregulated cell cycle gene sets from the TCGA database [31]. Analyses of our TNBC-enriched Guy’s cohort and publicly available TCGA and METABRIC datasets based on IHC-defined subtypes confirmed that cell cycle CDKs and cyclins were significantly upregulated in TNBC. Among those genes, cyclin D, the partner of CDK4/6, were significantly downregulated in TNBC compared to non-TNBC. Cyclin D downregulation can reduce the G1-to-S phase transition [58], contribute to breast cancer metastasis by increasing migratory capacity, and is associated with a poor prognosis [59]. Basal-like/TNBCs do not depend on the cyclin D/CDK4/6 complex to initiate cell cycle but may rely on cyclin E and CDK2. The cyclin E/CDK2 axis is known to enhance tumor growth in addition to CDK4/6 function, which subsequently drives cell cycle progression and could potentially bypass CDK4/6 inhibition for cell cycle transition [19]. Our analyses also confirmed previous findings that the high expression of cyclin E and CDK2 correlated with a worse overall survival in breast cancers [60].

Since TNBC lack targetable receptors for established treatments, such as endocrine or HER2 therapies, combinations of conventional cytotoxic chemotherapeutic agents remain the standard systemic therapy, although these tumors often develop chemoresistance [61]. The pan-CDK inhibitor dinaciclib alone or in combination with an AKT inhibitor shows a growth inhibitory effect in *CCNE1*-amplified cancers in vivo [62,63]. It has been suggested that CDK2 inhibition would target the reliance of poor prognosis breast cancers, including TNBCs, on cyclin E/CDK2 activation [19,23]. Presently, there is no CDK2 inhibitor approved for the treatment of breast cancer, but several novel drugs are currently undergoing pre-clinical evaluation against breast cancer [64,65,66]. To our knowledge, due to the structural homology between CDK proteins, it is challenging to identify CDK2-specific small-molecule inhibitors that do not possess some affinity for other kinases [67,68]. A highly specific CDK2 inhibitor is not yet commercially available, although one compound, namely PF-07104091, is under investigation in a phase I clinical study as a single agent, and in combination therapy for several cancer types, including TNBC (clinicaltrials.gov identifier: NCT04553133). Since there were no publicly available CDK2-specific inhibitors, in our study, we compared the inhibitory effect of three pan-CDK-targeting inhibitors (SNS-032, K03861 and JNJ-7706621). These inhibitors share the common property of exerting significant blocking effects on CDK2 among them (see Figure 2A Table). Our aim was to select a potent drug for subsequent cellular and in vivo studies. While the TNBC cell lines MDA-MB-231 and MDA-MB-468 were both sensitive to all inhibitors in a dose-dependent manner, SNS-032 demonstrated a superior efficacy. SNS-032 inhibition is thought to cause cell cycle arrest, inhibit transcription, and trigger cancer cell apoptosis [57]. SNS-032 was originally designed to be a selective CDK2 inhibitor [43]. However, it is also reported to block the activity of RNA Polymerase II by inhibiting CDK7 and CDK9 [42]. Our findings indicate that these two CDKs and their binding partners (cyclin H or cyclin T1) are downregulated in basal-like/TNBC. Thus, further studies are required to evaluate the precise targets of SNS-032 that drive the effect of the drug observed in the models of TNBC in the present study. SNS-032 was safely administered on a weekly intravenous schedule in the phase I clinical trial; however, the maximum tolerated dose (MTD) was not reached because the sponsor discontinued the study due to a change of priorities [69]. Another phase I clinical study reported myelosuppression as the most commonly observed toxicity, which was conservatively managed without dialysis or fatalities [42]. Both reports concluded that the drug could be evaluated at higher dosages in follow-up trials.

Approximately 35% of TNBC demonstrate a detectable expression of PD-L1 by cancer cells [70]. PD-L1 expression peaks at the M and early G1 phases and decreases at the late G1/S phase [18]. PD-L1 expression is upregulated in response to inflammatory cytokines such as IFN-γ [46], or in response to the immunosuppressive cytokine TGF-β expressed by cancer cells and recruited Tregs in the TME. Tregs can further produce immunosuppressive mediators such as IL-10, which can also promote PD-L1 expression [71]. The expression of PD-L1 by cancer cells and engagement with PD-1 in T cells can result in T cell anergy, apoptosis, and therefore weaker anti-tumor immunity [72,73]. However, checkpoint inhibitor treatment, which is designed to interfere with these interactions, is limited as a monotherapy for immunogenic tumors such as melanoma [74]. Poor responses in breast cancer patients may be due to several mechanisms, including relatively low PD-L1 expression, low immune cell infiltrates or low neoantigen load and insufficient corresponding cancer antigen-reactive immune responses [11,12,13,14]. Potentially, therapy with agents that promote pro-inflammatory signals or cancer cell apoptosis may offer some merits in TNBC, where tumor cell debris and danger signals from stressed and dying cancer cells may trigger the recruitment of lymphocytes into the TME. For example, dinaciclib, a potent CDK1/2/5/9 inhibitor, has been shown to induce apoptosis of cancer cells, and its combination with checkpoint immunotherapy resulted in an enhanced anti-tumor response [75]. Another example may be the combination of chemotherapy with atezolizumab for advanced-stage TNBC [3,4]. Short-term doxorubicin and cisplatin induction treatments in the TONIC trial demonstrated an upregulation of immune-related genes involving the PD-1–PD-L1 axis and T cell cytotoxicity pathways, which indicated a more favorable outcome for PD-1 blockade in TNBC [17]. Therefore, it may be beneficial to study targeted drugs that are able to prime the TME and be applied alongside immunotherapy.

The inhibition of CDK4/6 with palbociclib has been reported to stimulate PD-L1 expression in cancer cells by inhibiting ubiquitination-mediated PD-L1 degradation [18,21]. In our study, we found that SNS-032 treatment upregulated PD-L1 in some TNBC in vitro cellular and in vivo human xenograft models. Previous studies reported the cytotoxic effects of SNS-032 in glioblastoma, chronic lymphocytic leukemia (CLL), acute myeloid leukemia (AML) and multiple myeloma (MM) cells [44,76,77]. Approximately a 50% disease reduction was demonstrated in a phase I pharmacologic study of SNS-032 treating patients with CLL and MM, with reported toxicity associated with myelosuppression detected at the maximum-tolerated dose of SNS-032 [44]. These suggest that dosage selection for SNS-032 may be considered to avoid adverse cytotoxic effects, and treatment regimens could be designed to take advantage of CDK inhibition, whilst reducing the effective dose of inhibitor required to achieve a therapeutic benefit. A previous report demonstrating that CDK2 inhibition can restore chemosensitivity in TNBC cell lines also points to the merits of evaluating the potential therapeutic benefits of targeting CDK2 in a combination with other treatments [64]. In our study, we interrogated MDA-MB-231 xenografts in mice which we showed to be sensitive to SNS-032 inhibition and to upregulate PD-L1 by cancer cells in response to CDK inhibition. Since the inhibitor can be toxic at efficacious levels, we injected a relatively low and less frequent dosage of SNS-032 compared to previous in vivo studies (once weekly intravenous 15 mg/kg versus previously reported intraperitoneal doses of 15–30 mg/kg every 3 days) [47,48]. No mice treated at this concentration demonstrated any overt toxic effects from SNS-032 treatment. However, a moderate restriction of tumor growth was measured with these suboptimal drug levels. Alongside the moderate restriction of human TNBC xenograft growth, the suboptimal dosing of SNS-032 had two further effects: a) higher human CD45^+^ immune cell infiltration in human TNBC xenografts from immunodeficient mice engrafted with human immune cells, when compared with vehicle-treated controls and b) an upregulation of PD-L1 by tumor cells in vitro and in the TNBC model in vivo.

With regards to our flow cytometric data showing enhanced human lymphocyte presence in the TME following SNS-032 treatment in vivo, it is possible that SNS-032 can lead to dysregulation of the cell cycle and a level of cytotoxicity of tumor cells, where dead cell debris and danger signals can promote tumor antigen presentation, resulting in immune cell activation and enhanced immune surveillance. The CDK targeting of cancer cells may also stimulate the recruitment of immune cells into tumors by the induction of PD-L1 expression and other stress signals. On the other hand, anti-PD-L1 treatment appeared to reduce human CD45^+^ immune cell infiltration in tumor xenografts, and this may be due to impairing the proliferation of PD-L1 expressing human immune cells including by antibody effector mechanisms, as previously described [56]. Human immune cell infiltration into xenografts partly recovered with the addition of SNS-032 to avelumab treatment, further supporting the possibility that SNS-032 is responsible for promoting a level of immune cell recruitment into tumors.

With regard to our observation that suboptimal levels of SNS-032 inhibitor also upregulated PD-L1 expression by the surviving TNBC cells, it is possible that this inhibitor can exert pro-tumoral effects, by increasing the chance of PD-L1 expressing breast cancer cells to engage with immune cells in tumors and impair their activation. On the other hand, the expression of PD-L1 by tumor cells may mark these cells for targeting with an anti-PD-L1 antibody. In human-TNBC-xenograft-bearing mice engrafted with human immune cells, SNS-032 treatment, ahead of weekly dosing with the anti-PD-L1 antibody avelumab, led to the growth suppression of human TNBC xenografts, compared to either SNS-032 or avelumab monotherapies. Treatment with avelumab may also have several effects. These may include the blockade of the PD-1/PD-L1 axis between immune cells and cancer cells, reducing the suppression of PD-1 expressing immune cells. Furthermore, PD-L1 expression by cancer cells may allow an Fc-active anti-PD-L1 antibody such as the IgG1 isotype avelumab to engage immune effector cells in the TME via Fc-FcR interactions, to affect a level of cytotoxic killing of PD-L1-expressing cancer cells. A previous study demonstrated that avelumab can trigger ADCC by NK cells against tumor cells, alongside a checkpoint blockade by targeting PD-1 interactions with PD-L1 [49]. Here, we also confirmed the ability of avelumab to trigger NK-cell-mediated ADCC of PD-L1-expressing, but not of non-PD-L1-expressing, TNBC cells, at least in vitro. In vivo, it is possible that SNS-032-promoted upregulation of PD-L1 by surviving cancer cells may: (a) restrict the activation of immune cells recruited to the TME via the PD-L1-PD-1 axis, and (b) simultaneously allow avelumab to function against PD-L1-expressing cancer cells via effector functions or by the disruption of the PD-1-PD-L1 axis to reverse immunosuppression. It is possible that a combination of these mechanisms may contribute to the effects of treatment with SNS-032 followed by anti-PD-L1.

## 5. Conclusions

We evaluated the ability of the CDK inhibitor SNS-032 to impair TNBC cell survival and growth, and we demonstrated the elevation of PD-L1 expression by surviving cancer cells after treatment with suboptimal doses of SNS-032 in vitro and in a human xenograft model of triple-negative breast cancer. SNS-032 treatment was associated with the infiltration of CD45+ human immune cells into human TNBC xenografts in the mammary fat pads of immunodeficient mice that were engrafted with human immune cells. These findings may point to an association of CDK inhibition with both PD-L1 regulation on tumor cells and immune responses. In human-TNBC-xenograft-bearing mice given human immune cells, weekly treatment with SNS-032 followed by the anti-PD-L1 antibody avelumab demonstrated a reduction in tumor growth, compared with SNS-032 inhibition only or with anti-PD-L1 antibody alone, in the absence of overt toxic effects. Our data suggest that targeting breast cancers with suboptimal doses of a CDK inhibitor may promote immune cell infiltration, upregulate PD-L1 in surviving cancer cells, and potentially be considered alongside checkpoint blockade. Further studies are required to ascertain whether lower doses of CDK inhibitors can allow anti-PD-L1 treatment for TNBC that previously did not meet the threshold of PD-L1 expression for treatment with immunotherapy.

## Data Availability

For original data, please contact the corresponding author.

## Supplementary Materials

The following supporting information can be downloaded at: https://www.mdpi.com/article/10.3390/cancers14143361/s1, Supplementary Table S1. Summary of all internal and external cohorts used. Supplementary Figure S1. Gene expression level of transcription regulators in breast cancers. Supplementary Figure S2. Gene expression level of early phase cell cycle regulators in breast cancers. Supplementary Figure S3. High cyclin E and CDK2 expression in breast tumors compared to normal tissues. Supplementary Figure S4. Gene expression level of CDK7/cyclin H and CDK9/cyclin T in breast cancers. Supplementary Figure S5. PD-L1 assessment in TNBC cells in preparation for in vivo combination therapy experiments. Supplementary Figure S6. MDA-MB-231 xenograft tumor engraftment of human CD45^+^ immune cell population.
