# Supplementary material for: CDK Inhibition Primes for Anti-PD-L1 Treatment in Triple-Negative Breast Cancer Models
Source: Cancers (Basel). 2022 Jul 11;14(14):3361. doi: 10.3390/cancers14143361 (PMC9322647; doi:10.3390/cancers14143361)
Supplement: Supplementary file 1 [file cancers-14-03361-s001.zip › cancers-1712309-supplementary.pdf]

**Transcriptomic data:**<br>*Guy's, TCGA, METABRIC gene expression cohorts*<br>**(Fig. 1A, 1B, 1C, 5D; Suppl. Fig. 1, 2A, 2B, 4A, 4B)**<br><br>GEPIA cohort (Tang *et. al.*)<br>**(Suppl. Fig. 3Ai, 3Bi, 4C)**	**Samples**:<br>Guy's: primary TNBC, n = 131; non-TNBC, n = 46; normal n = 10<br>TCGA: primary TNBC,n = 123; non-TNBC, n = 515; normal, n = 112<br>METABRIC: primary TNBC, n = 101; non-TNBC, n = 1096<br><br>GEPIA: primary breast cancer, n = 1085; normal breast, n = 291
**Survival data:**<br>*KM plotter cohort*<br>(https://kmplot.com/; Györffy *et. al.)* **(Fig. 1D)**	**Samples:**<br>Primary breast cancer, n = 1402
**Protein expression data:**<br>*Human Protein Atlas cohort*<br>(www.proteinatlas.org) Uhlen *et. al.* **(Suppl Fig. 3Aii, 3Bii)**	**Samples:**<br>24 normal human organs (immunohistochemical stainings of tissue sections from one to three healthy volunteer donors).
**Cell line mRNA expression data:**<br>*Cancer Cell Line Encyclopedia (CCLE)*<br>(https://sites.broadinstitute.org/ccle) Barretina *et. al.* **(Fig. 2B)**	**Samples:**<br>CCLE database was used to characterize mRNA expression of *CDK2* in the eight TNBC cell lines used in this project.

Supplementary Table S1. Summary of all internal and external cohorts used.

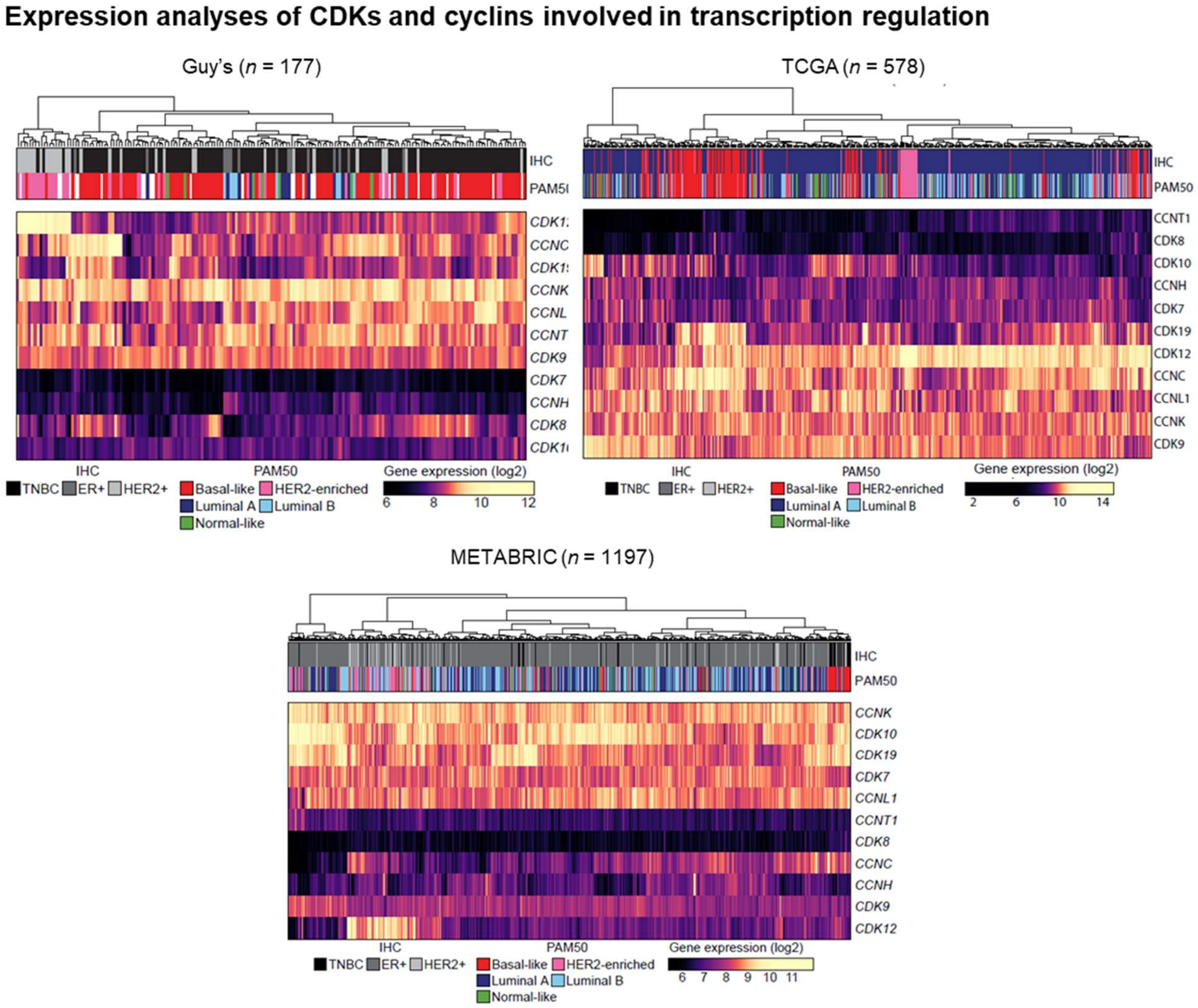

Supplementary Figure S1. Gene expression level of transcription regulators in breast cancers.

Gene expression analysis of primary tumors from Guy's (TNBC-enriched, n = 177), TCGA (n = 578) and METABRIC (n = 1197) cohorts of CDK and cyclin genes involved in transcription regulation. Color scale indicates log2 expression values (yellow, higher; black, lower expression). Cohorts were divided into TNBC, ER+ and HER2+ groups based on their IHC-defined receptor status, in addition to PAM50 classification (Basal-like, HER2-enriched, luminal A, luminal B and normal-like).

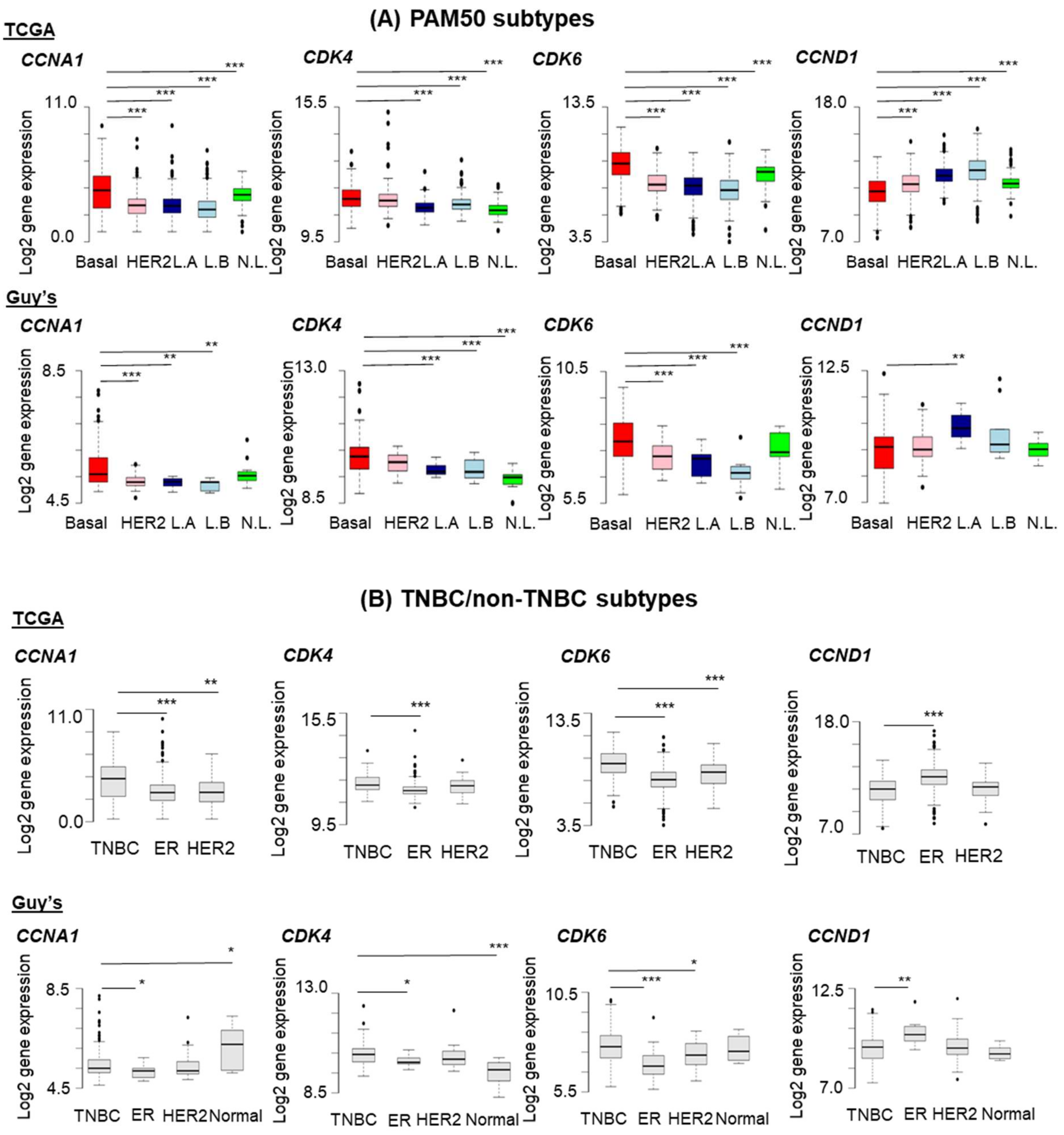

Supplementary Figure S2. Gene expression level of early phase cell cycle regulators in breast cancers.

Gene expression analysis of early phase cell cycle regulators in TCGA and Guy's cohorts classified by (A) PAM50 or (B) TNBC vs non-TNBC subtypes. Same as cyclin E and CDK2, the other early phase cell cycle regulators cyclin A (*CCNA1*), CDK4 (*CDK4*), CDK6 (*CDK6*) are all upregulated in basal-like/TNBC patients, although cyclin D (*CCND1*) were downregulated. All *p*-values were reported with the following associated symbols: $p < 0.05$ (*), $p < 0.005$ (**), $p < 0.0005$ (***), and all tests were two-sided.

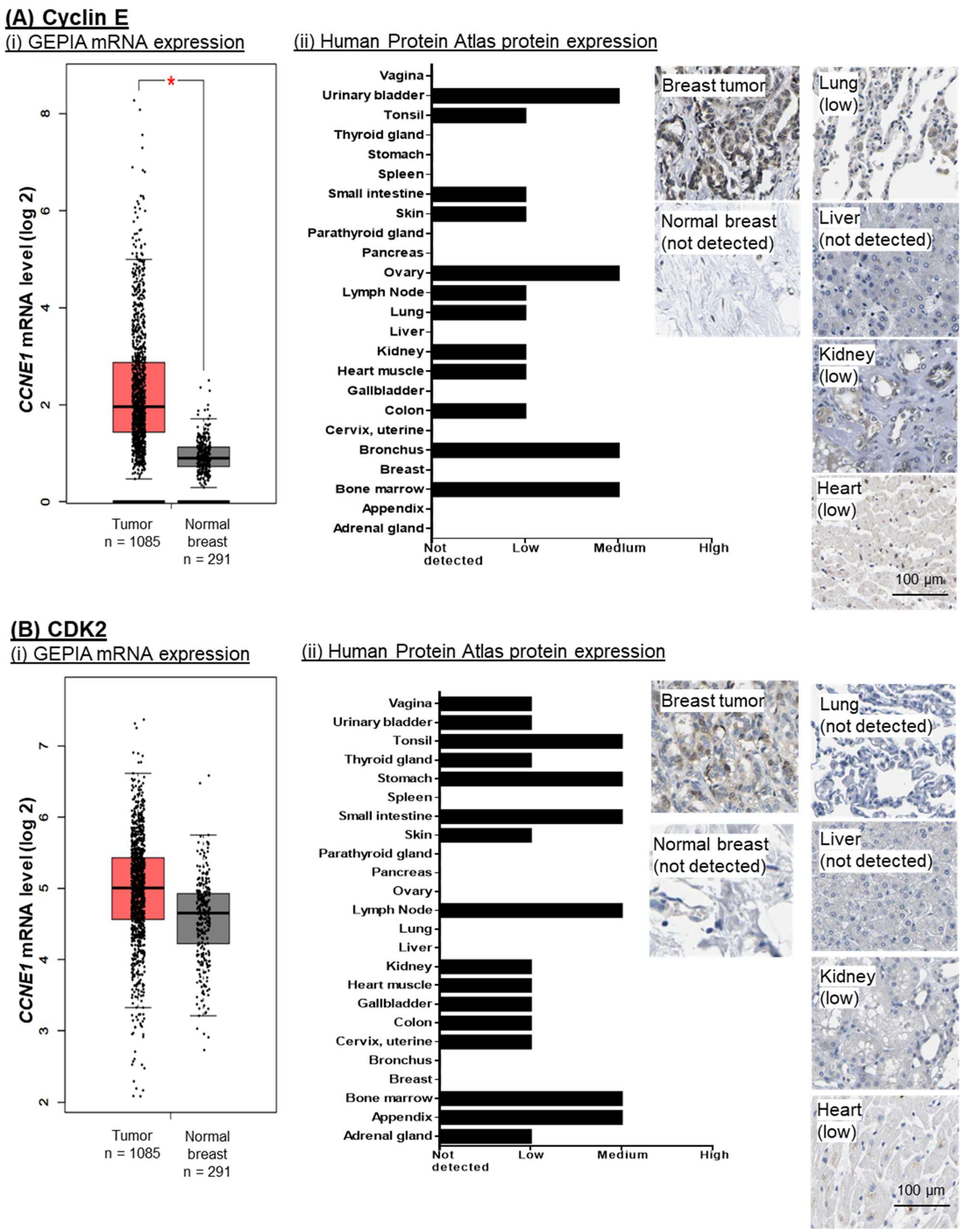

Supplementary Figure S3. High cyclin E and CDK2 expression in breast tumors compared to normal tissues.

To compare between breast tumors expression level of (A) cyclin E or (B) CDK2 with normal tissues, (i) GEPIA mRNA expression data are shown for primary breast tumors (n = 1085) and normal breast tissues (n = 291), and (ii) Human Protein Atlas histology-based protein

expression data are shown for major normal human tissues. Distribution of cyclin E and CDK2 in human organs are shown in 24 normal tissues. Immunohistochemical staining were performed from tissues of one to three healthy volunteer donors per tissue type, detection levels were classified as high, medium, low or not detected according to the staining intensity. Examples of the IHC-staining images are shown for vital human organs (lung, liver, kidney or heart). Data showed that both cyclin E and CDK2 were distributed in different organs mostly at low or medium levels (for cyclin E: 12 organs undetectable, 8 organs at low level, 4 organs at medium level; for CDK2: 8 organs undetectable, 10 organs at low level, 6 organs at medium level). All images are available from Human Protein Atlas version 19.3 (www.proteinatlas.org). Scale bar of representative images = 100 μm. All *p*-values were reported with the following associated symbols: $p < 0.05$ (*), $p < 0.005$ (**), $p < 0.0005$ (***), and all tests were two-sided.

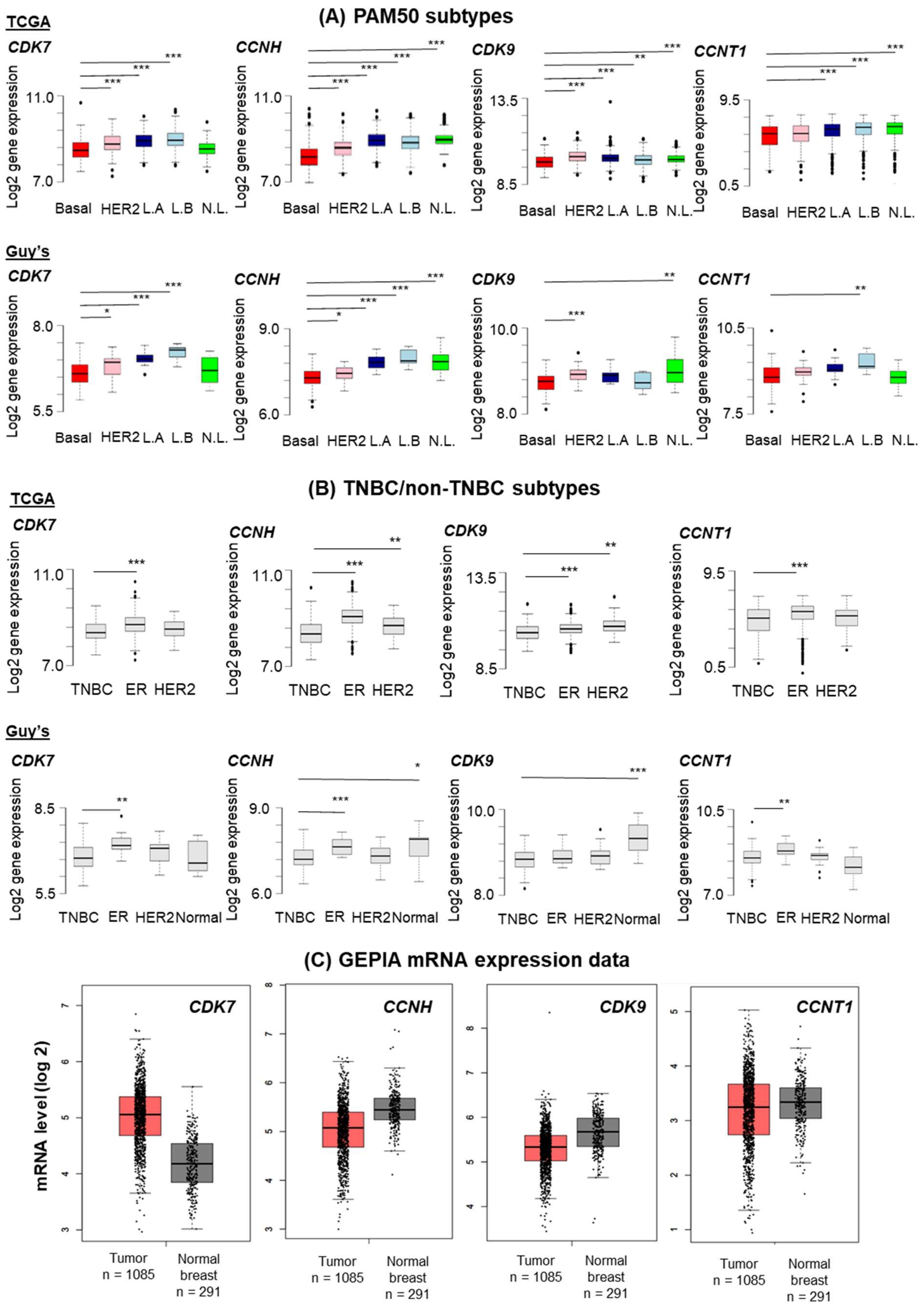

Supplementary Figure S4. Gene expression level of CDK7/cyclin H and CDK9/cyclin T in breast cancers.

SNS-032 inhibitor targeted CDK2, but also demonstrated activities against CDK7 and CDK9 in cell-free assay. Gene expression analysis of (A) PAM50 or (B) TNBC vs non-TNBC subtypes in both TCGA and Guy's cohorts showed downregulation of these two binding partners, CDK7/cyclin H and CDK9/cyclin T, in basal-like/TNBC patients, therefore no evidence of targeting these CDKs or cyclins for therapeutic strategies. (C) In addition, GEPIA data showed a small, but insignificant upregulation of CDK7 in breast tumors compared to normal breast tissues, however, cyclin H, CDK9 and cyclin T1 were all downregulated in breast cancers. Based on these data, normal tissue toxicity of SNS-032 should be low for those breast cancer patients, especially TNBC patients, with the downregulation of these genes. All *p*-values were reported with the following associated symbols: $p < 0.05$ (*), $p < 0.005$ (**), $p < 0.0005$ (***), and all tests were two-sided.

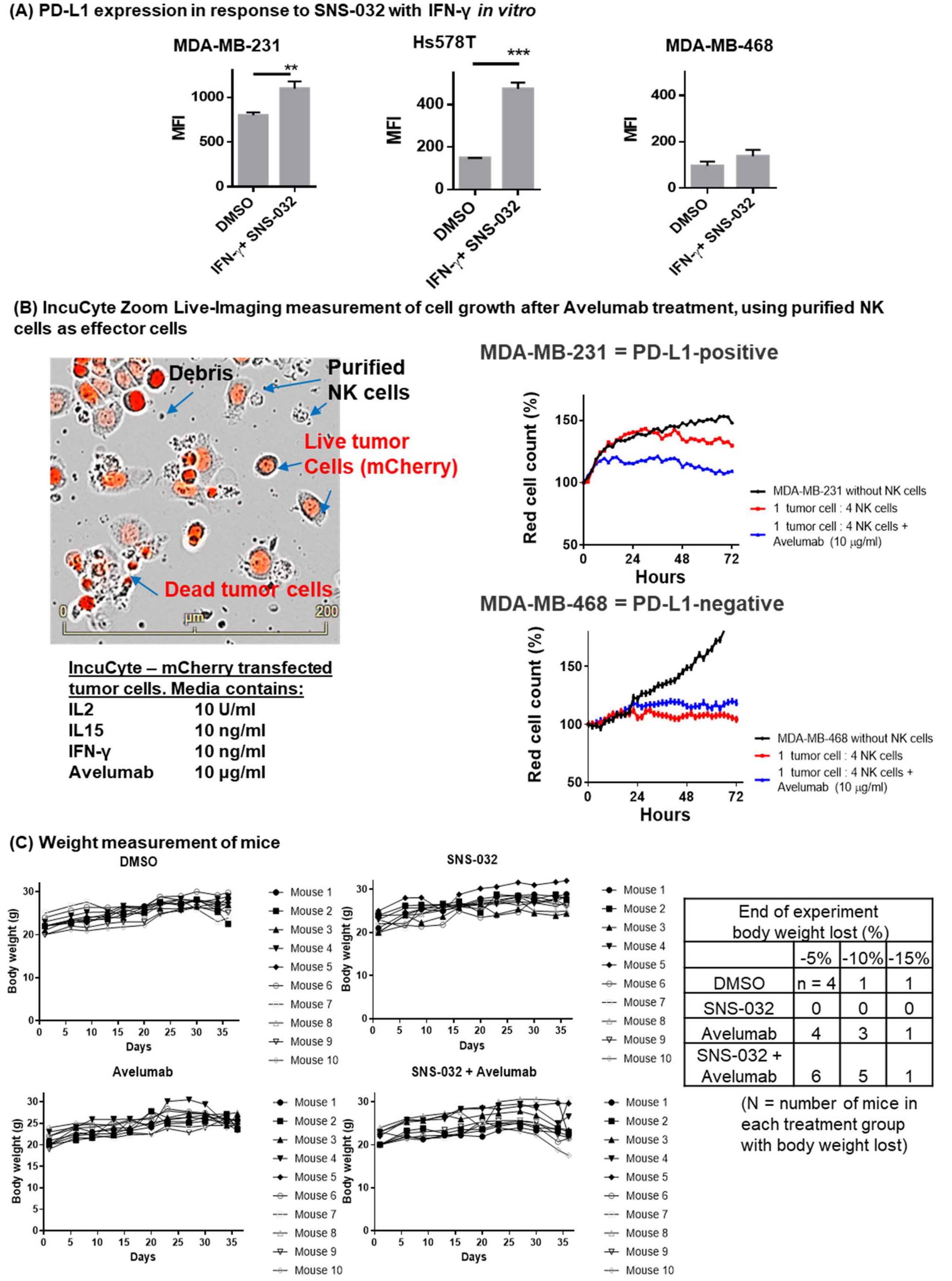

End of experiment body weight lost (%)			
	-5%	-10%	-15%
DMSO	n = 4	1	1
SNS-032	0	0	0
Avelumab	4	3	1
SNS-032 + Avelumab	6	5	1

(N = number of mice in each treatment group with body weight lost)

Supplementary Figure S5. PD-L1 assessment in TNBC cells in preparation for *in vivo* combination therapy experiments.

(A) Flow cytometry analyses of MDA-MB-231 and Hs578T demonstrated PD-L1 upregulation after treated with IFN-γ for 48 hours (n = 3). PD-L1 expression in MDA-MB-468 remained at background level. (B) Avelumab enhanced NK cell-mediated cytotoxicity in PD-L1 expressing TNBC cells and prevented cell proliferation. Avelumab effect was confirmed *in vitro* by IncuCyte Zoom Live-Imaging tracking of mCherry-transfected TNBC cell growth, using purified NK cells as effector cells (one cancer cells to four NK cells ratio). MDA-MB-231 cells expressing PD-L1 were sensitive to avelumab (10 µg/ml) and hence low cell count after 72 hours tracking, while PD-L1-negative MDA-MB-468 did not response to avelumab treatment. (C) Body weight measurement of the immunodeficient mice which received orthotopic (mammary fat pad) MDA-MB-231 cells together with human PBMC, and treated with SNS-032 or avelumab, or in combination, depicting lack of significant weight loss with tumor challenge, human immune cell engraftment and treatments. All *p*-values were reported with the following associated symbols: $p < 0.05$ (*), $p < 0.005$ (**), $p < 0.0005$ (***), and all tests were two-sided.

(A) Phenotyping of xenograft engrafted immune cells

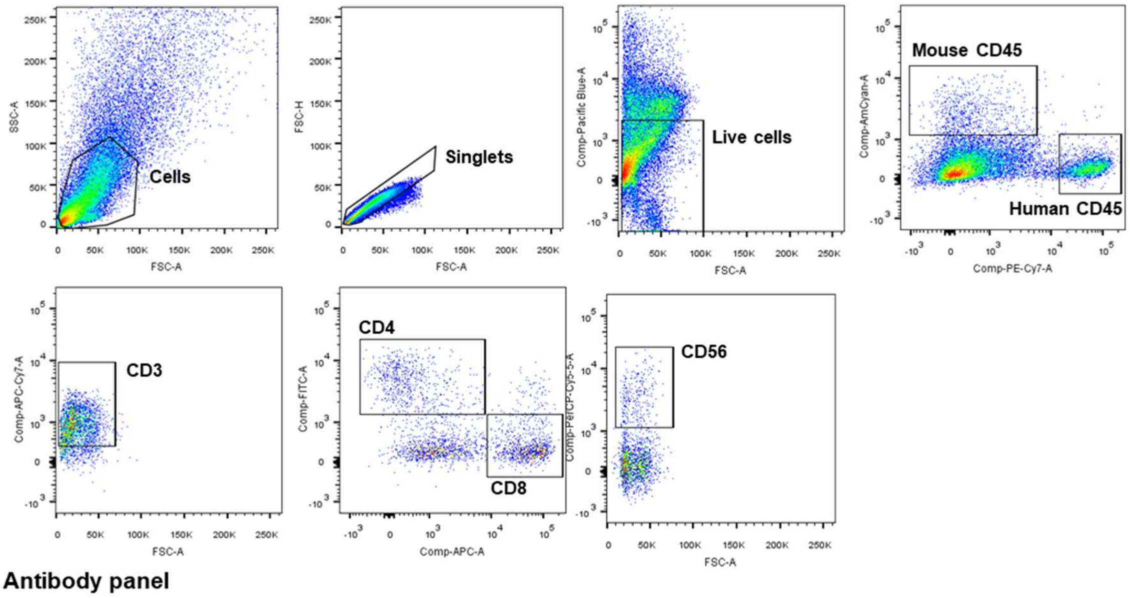

(B) Antibody panel

Reagent	Colour
Anti-mouse CD45	V500
Anti-human CD45	PE-Cy7
Anti-human CD3	APC-Cy7
Anti-human CD4	FITC
Anti-human CD8	APC
Anti-human CD56	PerCP-Cy5.5
Dead cell	DAPI

Marker	Cell type
CD45	Immune cells
CD3	T cells
CD4	T cells
CD8	T cells
CD56	NK cells

(All antibodies were purchased from Cambridge Bioscience)

(C) Xenograft tumor engraftment of human CD45^{+} immune cells comparison

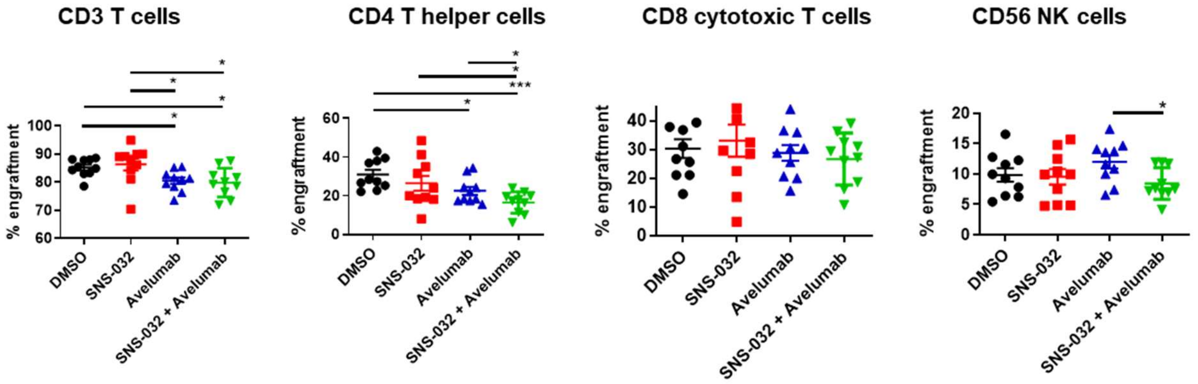

Supplementary Figure S6. MDA-MB-231 xenograft tumor engraftment of human CD45^{+} immune cell population.

(A) Gating strategies used in flow cytometry analysis for human immune cell engraftment in MDA-MB-231 xenograft model injected with human PBMC. Flow cytometric gating excluded any dead cells and cell aggregates which could increase autofluorescence and non-specific antibody staining. (B) Antibodies used for phenotyping were: rat anti-mouse CD45-V500; mouse anti-human CD45-PE-Cy7, CD3-APC-Cy7, CD4-FITC, CD8-APC and CD56-PercP Cy5.5. (C) Human immune cell engraftment (CD3 T cells, CD4 T helper cells, CD8 cytotoxic T cells and NK cells) in mouse tissues was calculated using the formula: % engraftment =

human immune cell population/human $CD45^+$ cells x 100%. All *p*-values were reported with the following associated symbols: $p < 0.05$ (*), $p < 0.005$ (**), $p < 0.0005$ (***), and all tests were two-sided.
